# An Exploratory Investigation on the Use of Closed-Loop Electrical Stimulation to Assist Individuals with Stroke to Perform Fine Movements with Their Hemiparetic Arm

**DOI:** 10.3389/fbioe.2016.00020

**Published:** 2016-03-07

**Authors:** Brian Lew, Nezam Alavi, Bubblepreet K. Randhawa, Carlo Menon

**Affiliations:** ^1^MENRVA, School of Engineering Science, Simon Fraser University, Burnaby, BC, Canada

**Keywords:** electrical stimulation, FES, stroke, functional movements, upper limb

## Abstract

Stroke is the leading cause of upper limb impairments resulting in disability. Modern rehabilitation includes training with robotic exoskeletons and functional electrical stimulation (FES). However, there is a gap in knowledge to define the detailed use of FES in stroke rehabilitation. In this paper, we explore applying closed-loop FES to the upper extremities of healthy volunteers and individuals with a hemiparetic arm resulting from stroke. We used a set of gyroscopes to monitor arm movements and used a non-linear controller, namely, the robust integral of the sign of the error (RISE), to assess the viability of controlling FES in closed loop. Further, we explored the application of closed-loop FES in improving functional tasks performed by individuals with stroke. Four healthy individuals of ages 27–32 years old and five individuals with stroke of ages 61–83 years old participated in this study. We used the Rehastim FES unit (Hasomed Ltd.) with real-time modulation of pulse width and amplitude. Both healthy and stroke individuals were tested in RISE-controlled single and multi-joint upper limb motions following first a sinusoidal trajectory. Individuals with stroke were also asked to perform the following functional tasks: picking up a basket, picking and placing an object on a table, cutting a pizza, pulling back a chair, eating with a spoon, as well as using a stapler and grasping a pen. Healthy individuals were instructed to keep their arm relaxed during the experiment. Most individuals with stroke were able to follow the sinusoid trajectories with their arm joints under the sole excitation of the closed-loop-controlled FES. One individual with stroke, who was unable to perform any of the functional tasks independently, succeeded in completing all the tasks when FES was used. Three other individuals with stroke, who were unable to complete a few tasks independently, completed some of them when FES was used. The remaining stroke participant was able to complete all tasks with and without FES. Our results suggest that individuals with a low Fugl–Meyer score or a higher level of disability may benefit the most with the use of closed-loop-controlled FES.

## Introduction

Stroke is the leading cause of upper limb disability and poor quality of life worldwide. Studies suggest that 3 months after stroke: 40% of stroke survivors suffer from significant upper extremity (UE), dysfunction of their affected arm, 40% have minor impairment, and only 20% retain full functionality (Buma et al., 2015). UE dysfunction includes motor deficits, functional deficits, and an inability to perform activities of daily living, thus increasing the burden of life (Feigin et al., 2008). Traditional rehabilitation techniques include high intensity-repetitive training, bilateral upper limb training, and constraint induced therapy to encourage neuroplasticity and early recovery (Intercollegiate Stroke Party, 2012). Functional electrical stimulation (FES) is a promising therapeutic treatment that complements the traditional therapy poststroke (Oujamaa et al., 2009). The most benefit seems to occur, however, when patients follow training schedules (Krakauer, 2006). Thus, to increase repetition and the efficacy of rehabilitation, the use of FES has been considered. FES allows the contraction of muscles independent of the central nervous system *via* electric current through surface or subcutaneous electrodes (Rushton, 1997).

Despite the beneficial results of FES, there is paucity of studies defining the detailed use of FES to achieve successful rehabilitation poststroke. Certainly, applying FES specifically to the limbs is challenging, because (1) commercially available stimulators employ an open-loop control, where the output movement is not fed back to the controller and (2) stimulator output has a pre-programed waveform of varying complexity, with no feedback or dynamic real-time alterations. On the other hand, graduated muscle contractions through closed-loop control may increase the precision, user safety, and robustness of FES because the output is modulated in real time according to a feedback loop (Zhang et al., 2013). A feedback loop could theoretically allow for finer control over the limb trajectory and thus ability to achieve complex maneuvers.

Even though there are numerous benefits, closed-loop FES systems are rarely available commercially, perhaps due to the challenge of implementing a control scheme that may be broadly applied to the non-linear and time varying behavior of muscles. Fatigue, spasticity, gravity, and training effects are other factors identified in distorting the controller performance (Ferrarin et al., 2001; Lynch and Popovic, 2012). In order to overcome these challenges, literature suggests different approaches. For example, Vette et al. (2007) and Sharma et al. (2012), suggested linear strategies with high gain feedback, but it cannot always guarantee system stability due to muscle non-linearity (Vette et al., 2007; Sharma et al., 2012). To compensate this flaw, machine learning (Vette et al., 2007) and iterated learning (Meadmore et al., 2014) algorithms have been applied. Other emerging promising non-linear strategies include sliding-mode control (Jezernik et al., 2004) and robust integral of the sign of the error (RISE) control (Sharma et al., 2009). Altogether, studies suggest that RISE control would be the preferred option of all due to the ease of implementation and stability. The RISE control guarantees stability under the assumption of a non-linear muscle model and appropriate controller gain constants (Sharma et al., 2009).

To the authors’ best knowledge, none of the studies have tested RISE methodology on hemiparetic arms in individuals with stroke. Hence, the first aim of this study was to assess the feasibility of applying closed-loop FES, utilizing the RISE controller algorithm during upper limb movements. We aimed to test it first in healthy individuals and next in individuals with stroke. We hypothesized that all participants (healthy and stroke affected) would be able to tolerate closed-loop FES. Tests with healthy subjects were first performed in order to verify that the tests could successfully be completed independently of the individual’s stroke condition. The healthy subjects verified the appropriateness of the procedure. These tests ensured that the potential inability of the individuals with stroke to complete tasks when assisted by FES was not inherently due to the adopted procedure.

Further, the second aim of this study was to assess the feasibility of using FES in individuals with stroke to augment their ability to perform functional tasks with their affected limb. Literature suggests that the majority of individuals with stroke develop abnormal flexion synergy in the UEs. It results in stereotyped, primitive mass movement pattern unsuitable to perform daily functional activities (Dipietro et al., 2007). For this study, stimulation of the affected side’s shoulder, elbow, and forearm muscles, namely, the anterior deltoid, infraspinatus, pectoralis major, biceps, triceps, and forearm extensor group, is hypothesized to aid participants to perform functional activities that are otherwise impossible because of this locked synergistic pattern poststroke.

## Materials and Methods

### Participants

Ethics approval for this study was obtained from the SFU Office of Research and Ethics. All participants provided informed written consent.

Four healthy individuals of ages 27–32 years old and five individuals with stroke of ages 61–83 years old participated in this study. The healthy individuals, seen in Table 1, had full functionality with both upper limbs and no history of neurological disorder.

**Table 1:** **Healthy participant data**.

Participant number	Sex	Age	Dominant hand
H1	M	27	R
H2	M	32	R
H3	M	29	R
H4	M	29	R
Mean (SD)	–	29.3 (2.1)	–

All stroke participants were screened to meet the following inclusion criteria: (a) age range from 39 to 85 years, (b) poststroke duration ≥6 months, (c) Montreal Cognitive Assessment (MoCA) ≥25 (Aggarwal and Kean, 2010), and (d) no history of shoulder dislocation. The exclusion criteria included (a) any other neurological conditions in addition to stroke, (b) unstable cardiovascular disease, (c) contraindications to FES, (d) history of arm pain, or (e) other conditions (e.g., poor sitting balance) that precluded them from undergoing the study. Further, we used the upper-extremity subtest of the Fugl–Meyer (FM) test to examine the impairment severity of all stroke participants (Gladstone et al., 2002). The FM scores of the participants ranged from 11 to 63, suggesting mild to severe motor impairments. The demographics and pre-assessment results of the participants are presented in Table 2. The severity of stroke for each of the participants was determined by using the following classification based on the FM score (Pang et al., 2006): severely impaired for 0–27 FM score, moderately impaired for 28–57 score, and mildly impaired for 58–66 score.

**Table 2:** **Stroke participant data**.

Participant number	Sex	Age	Months since stroke	Cognitive MoCA score	Dominant hand	Affected hand	Fugl–Meyer affected hand score
S1	M	67	47	25	R	L	49 (moderate)
S2	M	64	102	–	R	R	12 (severe)
S3	M	83	36	26	R	L	23 (severe)
S4	M	61	87	30	R	L	12 (severe)
S5	M	70	28	27	R	L	38 (moderate)
Mean (SD)	–	69 (8.5)	60 (32.6)	–	–	–	26.8 (16.4)

All participants signed the institution approved consent forms. One participant (S2) had expressive aphasia [15/30 on the Frenchay Aphasia Assessment (Al-Khawaja et al., 1996)] and was unable to complete the MoCA test. Nevertheless, he was still included in this study because he was able to follow the commands and complete the functional tasks. The Shapiro–Wilk test showed that the age, months since stroke, and FM assessment of the participants (see Tables 1 and 2) were normally distributed (α = 0.05).

### Apparatus and Control

**Figure 1:**
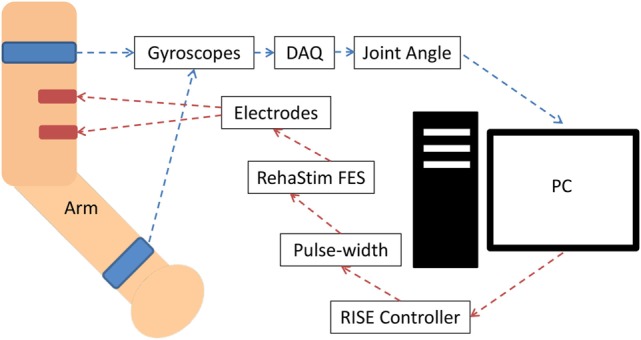
**System block diagram for biceps muscle stimulation**. The joint angle was calculated using two appropriately positioned strap-on gyroscopes, which communicated with the PC *via* a data acquisition device (DAQ) through USB. The PC communicated with the RehaStim device *via* a serial USB connection. Stimulation of other muscles had an analogous setup, but with alternate placement of the gyroscopes and electrodes.

The FES unit used in this study was the RehaStim I (Hasomed GmbH, Magdeburg, Germany), which has eight stimulation channels. RehaStim I has the capacity to generate biphasic rectangle pulses with a frequency range of 1–140 Hz, a pulse width range of 20–500 μs, and a current output range of 0–130 mA. For this study, we fixed the output frequency at 40 Hz but varied current amplitude between 10 and 30 mA based on the participant’s threshold. The modulation of both pulse width and amplitude of the output current was attempted in real time *via* serial USB communication with a computer as elaborated in Figure 1.

The pulse width was the control variable (CV) of the system and ranged between 20 and 500 μs. Joint angles were measured by attaching two multi-axes gyroscopes (Pololu LPR550AL) to the upper arm near the shoulder joint and the forearm near wrist as seen in Figure 1. For elbow extension/flexion corresponding to stimulation of the biceps or triceps, the elbow joint angle was measured. The degree of shoulder internal or external rotation was measured corresponding to stimulation of the pectoralis or infraspinatus. The degree of shoulder flexion was measured corresponding to the stimulation of the anterior deltoid. Lastly, the degree of the wrist dorsiflexion was measured corresponding to the stimulation of the forearm extensor. Joint angles were used as the process variables (PV), and a lowpass filter was employed to reduce the noise. The RISE controller algorithm was impemented by first calculating the error (*E*_1_) between the set point (SP) and PV:

$$E_{1}(t)=\text{SP}(t)-\text{PV}(t)$$

Following the RISE controller outlined by Sharma et al. (2012),

$$E_{2}(t)=\frac{d}{\mathrm{dt}}E_{1}(t)+\alpha_{1}E_{1}(t)$$

**Figure 2:**
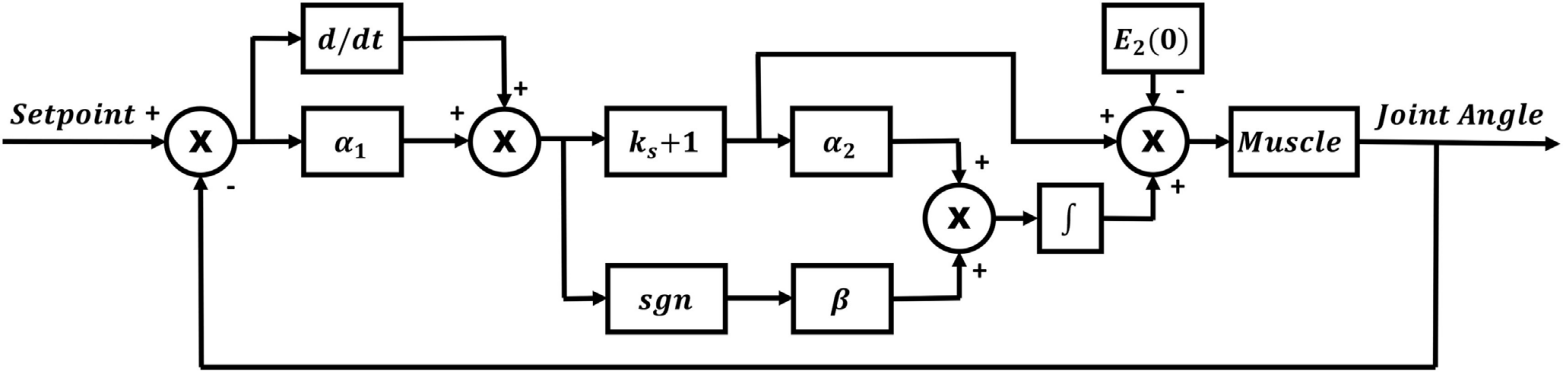
**RISE controller block diagram**.

The signum function, the defining feature of the RISE controller, was written as sgn in

$$\begin{array}{l} \text{PV}(t)=(k_{\text{s}}+1)E_{2}(t)-(k_{\text{s}}+1)E_{2}(0)\\ \;+\int_{0}^{\text{t}}\left[(k_{\text{s}}+1)\alpha_{2}E_{2}(\tau )+\beta \text{sgn}\left(E_{2}(\tau )\right)\right]\text{d}\tau \end{array}$$

where α_1_, α_2_, β, and *k*_s_ are adjustable, positive, control gain constants. These constants were adjusted individually based on the muscle being tested in every participant, since the tuning of a controller depends on the response of the plant. All muscles were first stimulated individually in separate trials, with the joint angle following a sinusoidal SP as seen in Figure 2.

### Experimental Protocol

For both healthy and stroke participants, individual muscles were first stimulated in separate trials, with the joint angle following a sinusoidal SP. The set of muscles included the anterior deltoid, infraspinatus, pectoralis major, biceps and triceps. These muscles facilitate the motions required for the functional tasks chosen, as well as being part of the UE FM assessment. As explained above, we chose these muscles because individuals with stroke follow a synergistic pattern of movements. Post stroke, majority of the times, the UE is locked in shoulder girdle retraction and elevation, shoulder abduction and external rotation, supination and flexion of the elbow, wrist, and finger flexion. Individuals with stroke need intense therapy to break this pattern and to re-learn the normal pattern of movements (Dipietro et al., 2007). In addition, these individuals need cues and mental training to control individual muscles sequentially to execute any functional task. Through FES, we can train the activation of specific muscle groups sequentially to perform meaningful movement.

For healthy participants, dominant (right) side and for stroke participants, the stroke-affected side was stimulated. Participants were instructed to sit on an armed chair in front of a table with equipment. All participants were requested to relax while using the FES equipment, to verify whether the robust control of the muscle using the RISE controller was possible, as well as to tune its gain constants. If successful with single joint movements, compound shoulder and elbow movements were attempted. Two muscles were stimulated simultaneously with the joint angles emulating a sinusoid over one cycle for the elbow joint and a ramp trajectory for the shoulder joint.

For the isolated muscle stimulation tests, the participants were positioned to conveniently test movements of their arm. The participants’ positions are schematically shown in Table 3. Both healthy and stroke individuals attempted compound motions to assess the feasibility of manipulating multiple joints simultaneously.

**Table 3:** **Details of initial and stimulation position with simulated isolated muscle trials side and top view**.

Muscle	Initial position	Stimulation assistance	Simulated isolated muscle trials with side and top view
Biceps	• Arm relaxed against the side of the body • Elb Flx = 0°	• Supination and Elb Flx = 45°	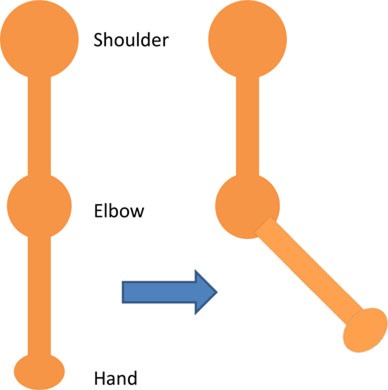
Triceps	• Upper arm supported with chair’s backrest to flex/extend elbow by 90° • Elb Extn = 90°	• FA Extn against gravity by 40°	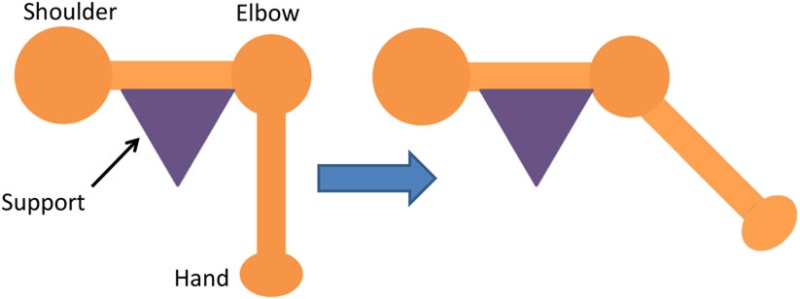
Pectoralis major	• Arm vertically aligned and relaxed against the side of the body • IR = 0°	• Achieve IR = 40°	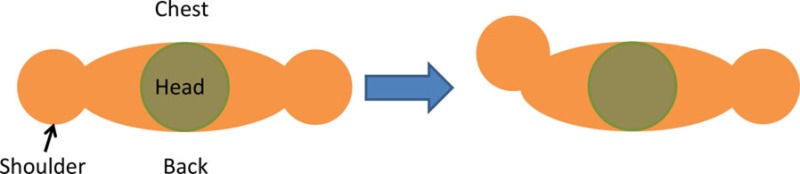
Infraspinatus	• Arm vertically aligned and relaxed against the side of the body • ER = 0°	• Achieve Sh ER = 40°	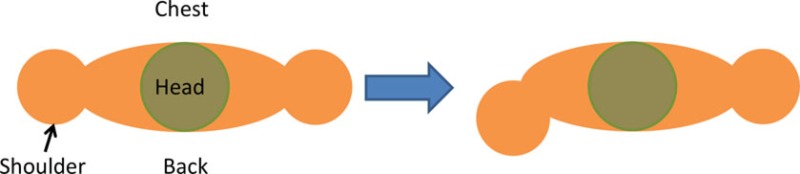
Anterior deltoid	• Arm vertically aligned and relaxed against the side of the body • Sh Flx = 0°	• Achieve Sh Flx = 40°	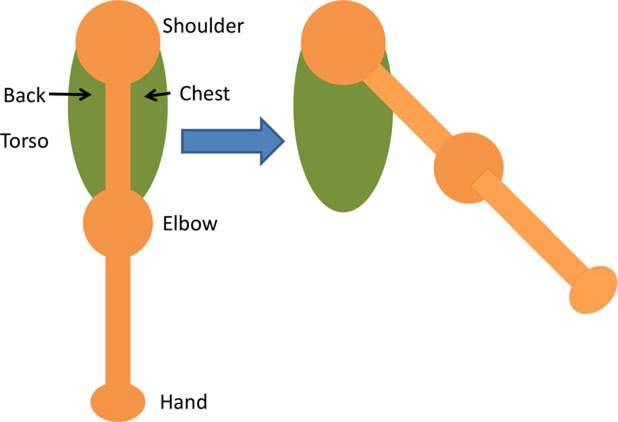

*IR, internal rotation; ER, external rotation; Sh, shoulder; Flx, flexion; Extn, extension; FA, forearm*.

Having assessed the feasibility of compound motions with healthy participants, individuals with stroke also attempted compound or single joint motions with their stroke-affected arm according to five functional tasks as explained in Table 4.

**Table 4:** **Functional task details of initial and stimulation position of muscles, including side, top, and front views**.

Muscles stimulated and task accomplished	Starting position	Stimulation assistance	Diagram
• Triceps • Picking up basket from the ground	• Seated with a basket on side at hand level • Arm relaxed against the side of the body	• Elb Extn to reach down and grasp the basket	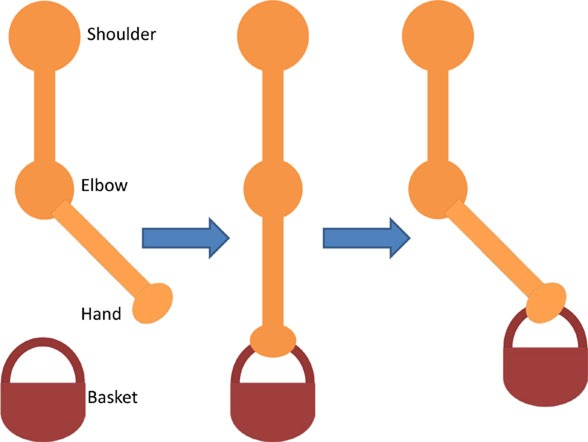
• Infraspinatus and biceps • Pick and place an object on a table	• Seated with forearm resting on the table • Elb = 90° Flx	• Elb Flx and Sh ER ~40° to pick and pace object to the side	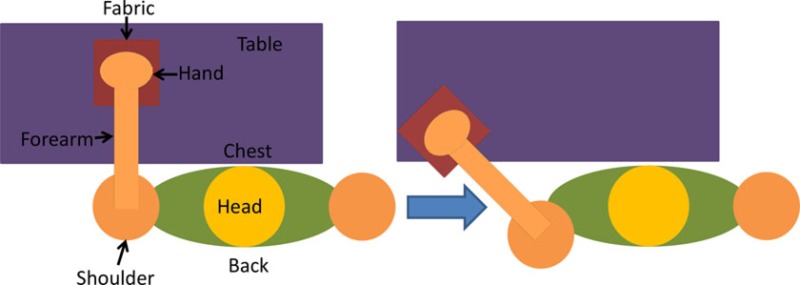
• Anterior deltoid • Cutting a pizza	• Upper arm against the side of the body • FA at an angle slightly below the horizontal holding knife in hand	• Sh flx to perform a single forward cutting motion	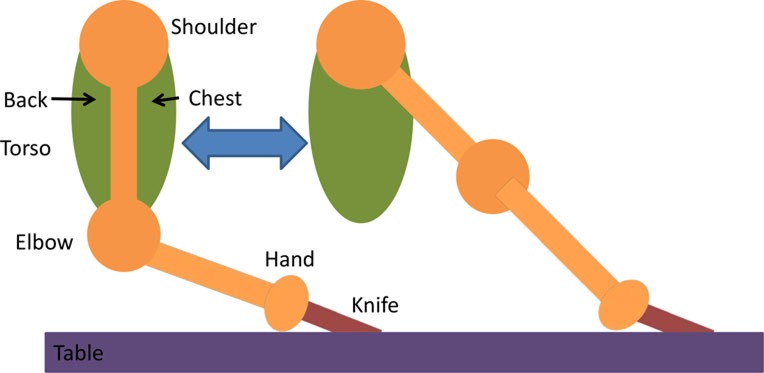
• Triceps and anterior deltoid • Pushing chair forwards	• Arm vertically aligned and relaxed against the side of the body	• Sh Flx and Elb Extn to push chair forwards and pull backwards	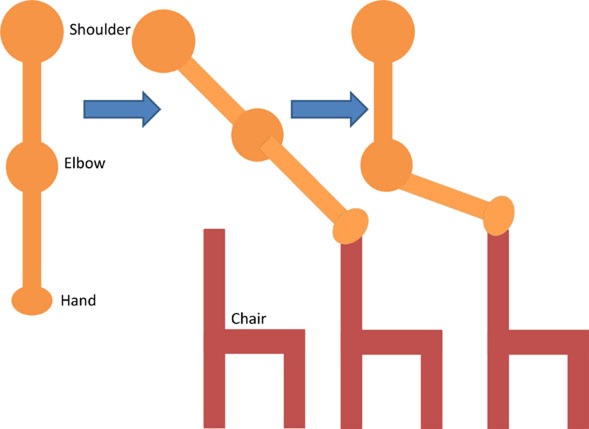
• Biceps and anterior deltoid • Eating with a spoon	• Hold a spoon while resting FA on a table	• Sh Flx and Elb Flx to bring the spoon to their mouth as if eating	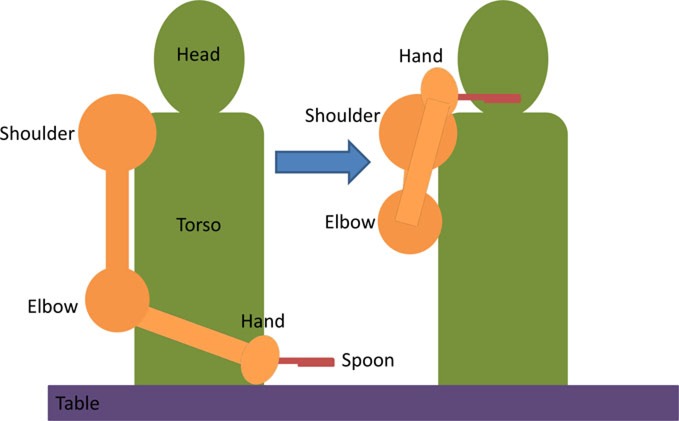

*ER, external rotation; Sh, shoulder; Elb, elbow; Extn, extension; Flx, flexion; FA, forearm*.

Two additional tasks were added to further challenge the motor skills of individuals with moderate/mild severity (FM > 28). In these tasks, the wrist extensor muscles were stimulated to achieve dorsiflexion according to the functional tasks presented in Table 5.

**Table 5:** **Details of the initial and active stimulation position for wrist dorsiflexion functional tasks, including side and top views**.

Task	Initial position	Final position	Diagram
Use a stapler	• Forearm and hand (facedown) resting on table	• Hand extension by 40° due to stimulation • Forearm moves toward stapler so that the hand rests on top of the stapler (gravity assisted)	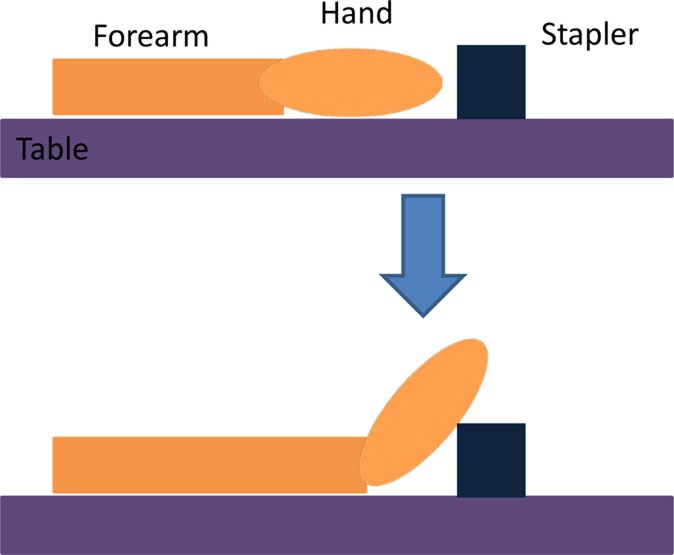
Grasp a pen	• Forearm and hand resting on table • Hand is oriented vertically on a table, with the thumb pointing up	• Wrist dorsiflexion by 50° due to stimulation, allows grasping of pen placed beside the palm at 50° extension	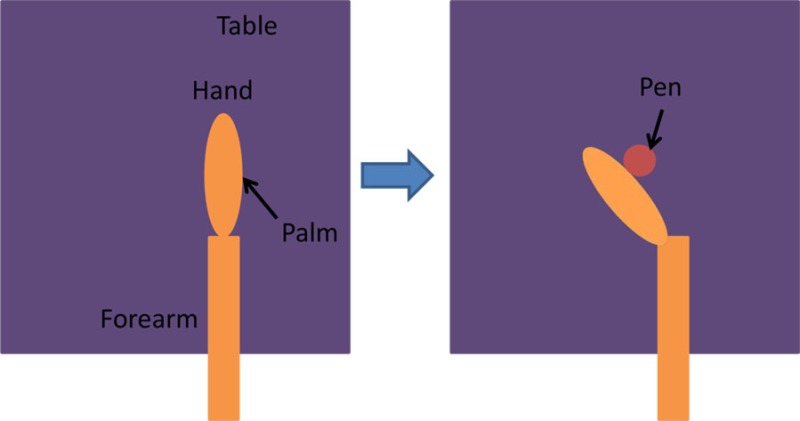

## Results

**Figure 3:**
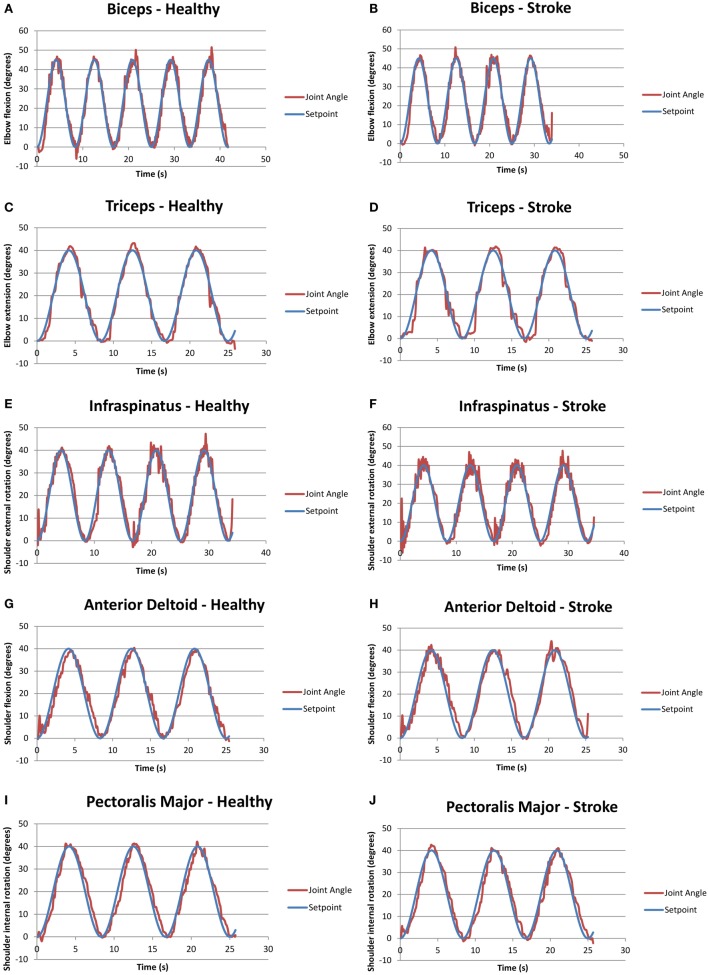
**Graphs of closed-loop FES when applied to various muscles for healthy and stroke individuals**. **(A)** Biceps for a healthy volunteer (H2); **(B)** biceps for a volunteer with stroke (S1); **(C)** triceps for a healthy volunteer (H2); **(D)** triceps for a volunteer with stroke (S2); **(E)** infraspinatus for a healthy volunteer (H4); **(F)** infraspinatus for a volunteer with stroke (S1); **(G)** anterior deltoid for a healthy volunteer (H4); **(H)** anterior deltoid for a volunteer with stroke (S5); **(I)** pectoralis major for a healthy volunteer (H4); and **(J)** pectoralis major for a volunteer with stroke (S1).

Figure 3 presents examples of FES assisted motions for healthy and individuals with stroke. It should be noted that healthy individuals were asked to keep their arm fully relaxed in these tests. Figure 3 shows that it is feasible to apply a closed-loop control *via* the RISE algorithm with a sinusoidal SP to various muscles in both healthy and stroke individuals. All healthy participants were able to follow the SP with varying degrees of success. Different gain constants and current amplitude levels were required for each participant and each muscle.

It should be noted that two stroke participants, namely, S3 and S4, were unable to follow some of the set points due to limited joint mobility and severe stroke impairment. Specifically, both stoke individuals were unable to follow the set points of the anterior deltoid and infraspinatus, and S4 was also unable to use the pectoralis major. Table 6 summarizes the success/failure for each stroke participant in following the sinusoidal SP for the isolated muscle trials. Green cells, also labeled “able follow,” indicate that the subject was able to follow the SP. Red cells, also labeled “failure,” indicate that the participant was not able to follow the SP.

**Table 6:** **Sinusoidal set point trial results for isolated muscles**.

Stroke participant	Biceps	Triceps	Pectoralis major	Infraspinatus	Anterior deltoid
S1	Able follow	Able follow	Able follow	Able follow	Able follow
S2	Able follow	Able follow	Able follow	Able follow	Able follow
S3	Able follow	Able follow	Able follow	Failure	Failure
S4	Able follow	Able follow	Failure	Failure	Failure
S5	Able follow	Able follow	Able follow	Able follow	Able follow

*Green indicates the subject was able to perform the task only when assisted by FES. Red indicates the subject was not able to perform the task weather assisted or not assisted by FES*.

Table 7 summarizes the calculated root-mean-squared (RMS) error for the angular range of the five muscle group trials conducted for healthy and stroke individuals, specifically for the corresponding trials as seen in Figure 3. Table 7 includes only data shown in green from Table 6.

**Table 7:** **RMS error of the trials with closed-loop FES on muscles**.

Muscle group	RMS error for healthy (°)	RMS error for stroke (°)
Biceps	3.64	4.49
Triceps	2.28	3.15
Pectoralis major	3.57	3.39
Infraspinatus	3.28	3.64
Anterior deltoid	2.16	3.67

**Figure 4:**
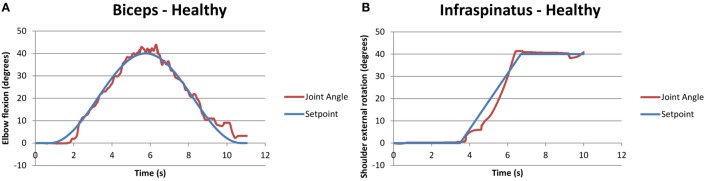
**Example of compound biceps (A) and infraspinatus (B) stimulation for a healthy participant, performing a motion analogous to pick and place for stroke participants**. The two motions occurred simultaneously.

Figure 4 shows an example of a functional task (pick and place an object) when the closed-loop control was used to assist multiple joint movements in a healthy volunteer (H4).

**Figure 5:**
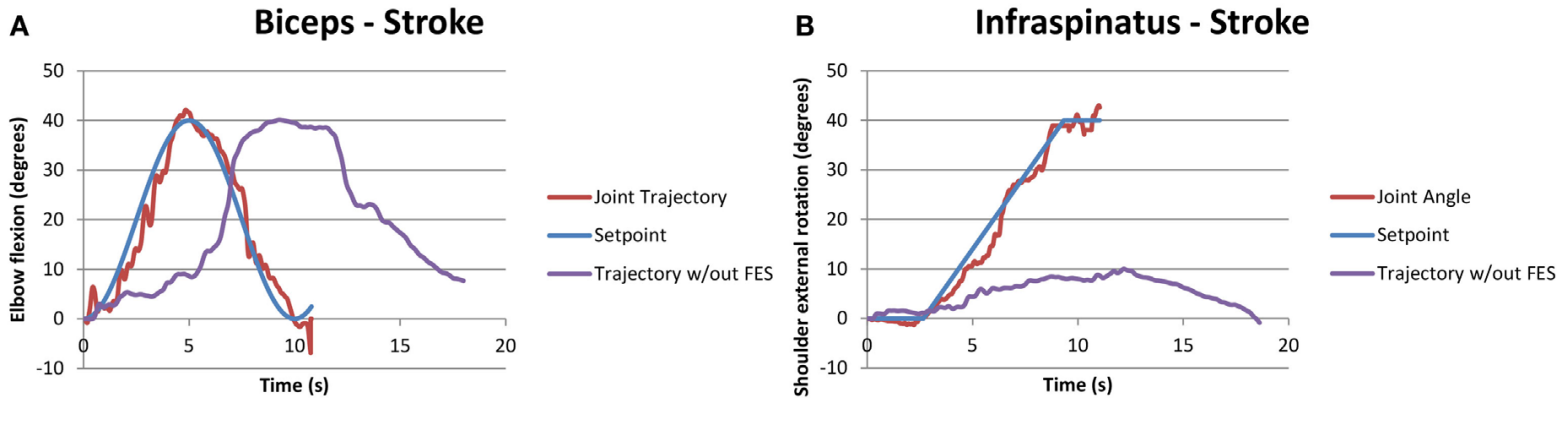
**Example of successful compound biceps (A) and infraspinatus (B) stimulation for a stroke participant performing the functional task “pick and place an object**.” The two motions occurred simultaneously, with the joint trajectory of the participant attempting the same task without FES also shown in purple for the infraspinatus.

Similarly, Figure 5 shows a compound motion for a stroke participant (S2). It should be noted that in this case, the participant could not rotate his shoulder externally with his affected arm, as shown by the purple line in Figure 5B, but he was able to follow the SP when FES was applied.

Table 8 summarizes the ability to perform functional tasks with and without FES for all participants with stroke. The color coding of Table 8 is as follows: yellow is used to indicate that participants were able to perform the tasks both independently and when assisted with FES, and is labeled “with and without FES”; red is used to indicate that participants could not perform the tasks with or without FES and is labeled “unable”; and green is used to indicate that participants were able to perform the task only when assisted with FES and is labeled “only FES facilitated.”

**Table 8:** **Summary of functional task improvement with FES in participants with stroke**.

Participant	Pick and place on table	Pick up basket from ground	Cutting pizza	Eating with spoon	Pull chair back	FM UE Scores
S1	With and without FES	With and without FES	With and without FES	With and without FES	With and without FES	49
S2	Only FES facilitated	Only FES facilitated	Only FES facilitated	Only FES facilitated	Only FES facilitated	12
S3	Unable	With and without FES	With and without FES	With and without FES	Unable	23
S4	Unable	Only FES facilitated	Unable	Unable	Unable	12
S5	Only FES facilitated	Only FES facilitated	With and without FES	With and without FES	Only FES facilitated	38

*Green indicates the subject was able to perform the task only when assisted by FES. Red indicates the subject was not able to perform the task weather assisted or not assisted by FES. Yellow indicates the subject was able to perform the task without FES assistance*.

S1 had a moderate level of stroke impairment and was able to perform all tasks without FES. S2 had a severe level of impairment with low FM score. He was unable to perform any of the functional tasks investigated in this study autonomously, but accomplished all tests with FES, despite his stroke severity. S3 was severely impaired and was able to perform 3/5 tasks with or without FES. This participant was unable to perform the other 2/5 tasks even with FES assistance, corresponding to failure in the anterior deltoid and infraspinatus isolated stimulation trials as seen in Table 6. This was likely due to a combination of the participant’s muscle and joint stiffness, owing to passive resistance from surrounding tissues or co-contraction of antagonistic muscle groups. S4 had a severely impaired arm and was only able to perform 1/5 tasks only with FES facilitation. Sensory impairment was likely the cause of failure for the other 4/5 tasks as well as 3/5 isolated muscle trials as shown in Table 6. This participant in fact complained of pain with any increase in amplitude to assist in task completion. S5 was moderately affected poststroke and he benefited from FES. He was able to perform 2/5 tasks independently and the other 3/5 with FES stimulation.

**Figure 6:**
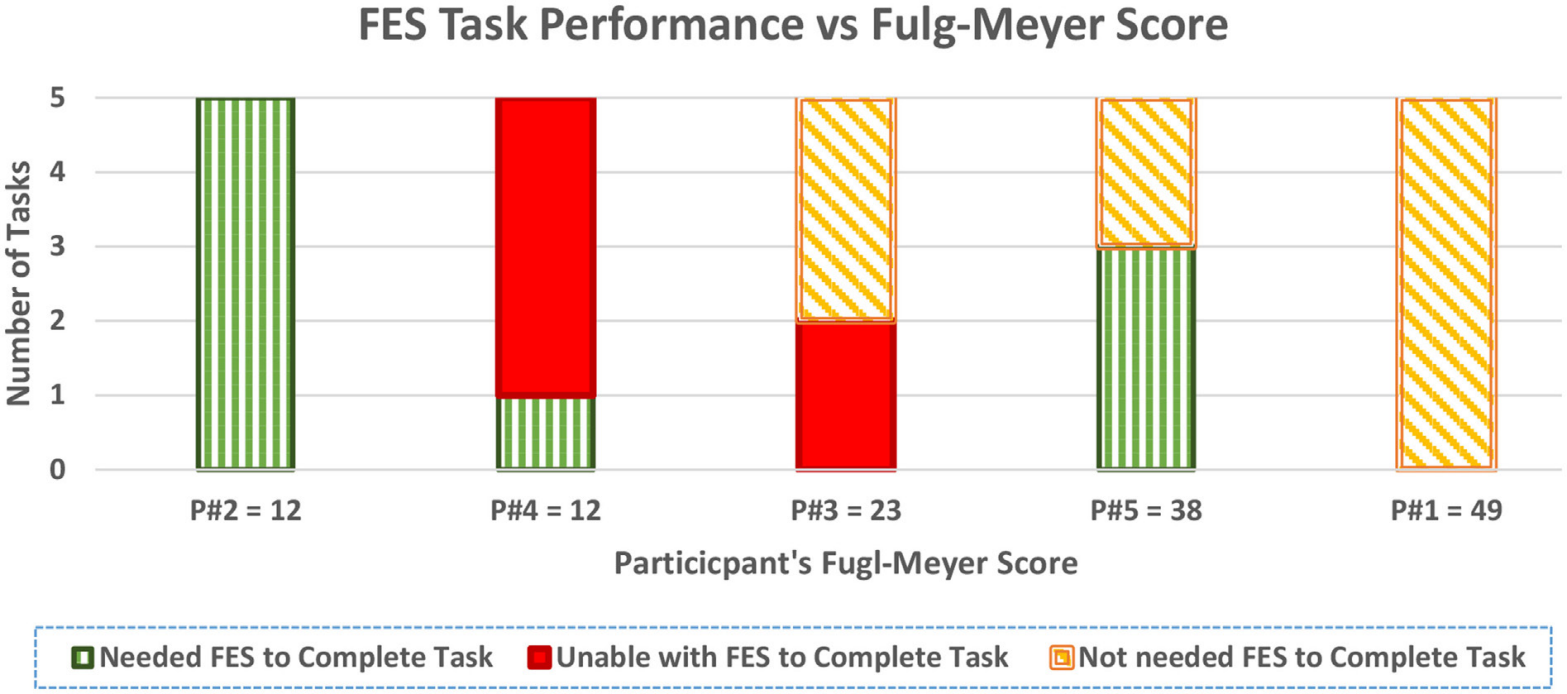
**Task assistance performance of FES with increasing Fugl–Meyer score**.

Figure 6 also shows both the performance of the FES system for each participant across the five tasks, and the FM score of each participant organized in an increasing order.

**Figure 7:**
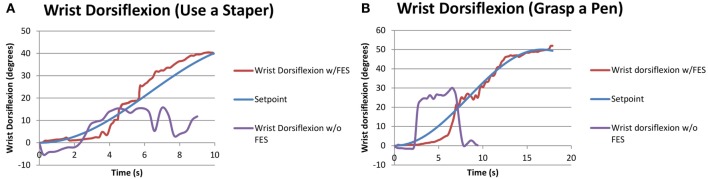
**Performance of functional tasks associated with wrist dorsiflexion through FES application**. The same subject attempting the same task without FES is also shown for comparison. **(A)** S5 doing “use a stapler” task. **(B)** S1 doing “grasp a pen” task.

Since subjects S1 and S5 had moderate/mild severity (FM > 28), they were asked to perform additional tasks that further challenged their motor skills (see Table 5). None of the two subjects were able to perform these additional tasks without FES assistance. Results for the performed tests, summarized in Table 9, show that the closed-loop approach proposed in this work was fully successful in this case. Figure 7 demonstrates how an FES application is able to increase wrist range of motion (ROM) and enable subjects to complete tasks that they cannot otherwise perform.

**Table 9:** **Summary of wrist dorsiflexion tasks**.

Participant	Use a stapler	Grasp a pen
S1	Only FES facilitated	Only FES facilitated
S5	Only FES facilitated	Only FES facilitated

*Green indicates the subject was able to perform the task only when assisted by FES*.

Generally, wrist ROM was greater in the trials with pen grasping compared to the stapler task (see Table 5), likely due to the orientation of the arm. During the stapler and pen tasks without the FES system, the participants were neither able to follow the set point of the stapler task nor the sinusoidal wave of reaching for the pen and returning back while grasping the pen. The participants could follow the desired motions and complete the functional tasks when assisted by FES.

## Discussion

The feasibility of using closed-loop FES to assist arm movements of stroke individuals was investigated in this study. We report that four of the five stroke participants benefited in functional task performance due to the application of FES, as seen in Table 8. In addition, two individuals with low FM scores (severe impairment) achieved the set points and performed functional tasks with FES. These are very exciting results demonstrating that FES with a closed-loop controller can be a potential tool for finely assisting functional tasks. It could increase the independence and confidence of stroke survivors by reducing dependence on caregivers.

Generally, the RMS error is indicative of the controller’s performance. Higher values reflect poorer tracking of the SP, while lower values reflect accurate tracking. It should be noted that the performance of the controller was noticeably dependent on the amount of time and degree of success in tuning the constants, which had high variability across the trials. The highest RMS error that was observed in both healthy and stroke individuals was the biceps muscle, may be because of the variation in origin and insertion of the muscle fibers. In addition, there was higher error in the performance of the pectoralis major muscles, which may be because it is a thick fan-shaped muscle situated at the chest, which may require different settings for appropriate stimulation. Due to these confounding factors (muscle shape, size, position, and orientation of muscle fibers), objective conclusions cannot be drawn on the controller performance comparisons between healthy and stroke participants. Overall, RMS error values for healthy and stroke participants were comparable to results from the same control scheme applied to stimulation of the quadriceps of unimpaired, healthy participants (Sharma et al., 2012).

For the functional tasks attempted in our study, individuals with stroke were instructed to relax the affected arm, except to activate appropriate forearm muscles when hand manipulation was required. From a controller perspective, it is unknown if a similar performance could be achieved if FES is working as a standalone system or when both FES and the required muscles are working together. One option is to have full reliance on the controller where the participant is relaxed and FES is working to achieve task completion. The second option would be to have FES supplement the participant’s own effort in order to complete a task. Future research may investigate if FES is helpful as a standalone modality (as used in this study) or if the FES system and the participant may work together, complementing each other during the active movement.

Literature suggests that despite of the intensive therapy in acute phase poststroke, few individuals with severe impairment are unable to re-gain motor control. These chronic stroke-affected participants suffer from weakness, spasticity, atrophy, and stiff joints due to stroke and learned non-use. FES could be beneficial for such individuals as our study results suggest. As seen in Figure 5B, one participant with stroke (S2) could not perform external shoulder rotation exceeding 10°, but FES increased their ROM, allowing external rotation up to 40° as required in activities of daily living. A few other participants with stroke had difficulty with full elbow extension and shoulder flexion – electrical stimulation applied to the triceps and anterior deltoid, respectively, provided the extra ROM to assist in tasks of daily living. Thus, the increase in ROM with FES could increase the quality of life and independence for individuals with stroke.

Results of this study show that the proposed FES closed-loop approach can be useful to assist some individuals with a hemiparetic arm in activities of the daily living requiring the simultaneous use of multiple arm joints. For instance, FES was very effective in assisting S2 who, despite his severe impairment in arm motor function, successfully completed all the assigned functional tasks when assisted by FES (Table 8). The proposed approach resulted to not be very suitable for individuals with severe motor impairments who did not tolerate well the current amplitude of FES, as in the case of S4, or had very stiff joints (difficult to move manually), as in the case of S3. For individuals with moderate/mild severity, such as S1 and S5, FES was obviously not useful to assist arm movements they could perform without any assistance (e.g., Table 8), but it was highly effective to assist in fine motor tasks, such as accurate wrist dorsiflexion movements (Table 9).

Thus, we conclude that FES can assist in the improvement of functional tasks for stroke survivors, but other factors, such as skin resistance, joint stiffness, and sensory impairment, can affect its usability. Therefore, a therapist should individually assess the usability of closed-loop controlled FES after thorough sensory and motor assessment of their participants.

This exploratory study showed that closed-loop-controlled FES can suitably recruit muscle groups to accurately follow the given trajectories for arm joints in both healthy and individuals with stroke. Closed-loop-controlled FES can assist individuals with stroke in functional tasks. While the current study purely focused on the arm assistance in functional tasks, future studies are warranted to investigate the use of the closed-loop-controlled FES as a standalone technology to improve the motor function of the hemiparetic arm of individuals with stroke.

## Author Contributions

BL: implemented the controller, collected data on stroke and healthy participants with the FES system, analyzed the data, and prepared the body of the manuscript. NA: collected stroke assessment data from participants, edited, modified, and finalized the manuscript. BR: assisted in the recruitment of stroke participants, assisted in designing tasks for the study, and edited the manuscript. CM: principal investigator, he conceived the closed-loop scheme, supervised the project, and contributed to design the study and prepare the manuscript.

## Conflict of Interest Statement

The authors declare that the research was conducted in the absence of any commercial or financial relationships that could be construed as a potential conflict of interest.
